# Inhibitory effects of iron depletion plus eribulin on the breast cancer microenvironment

**DOI:** 10.1186/s12885-020-07673-9

**Published:** 2020-12-10

**Authors:** Wataru Goto, Shinichiro Kashiwagi, Yuka Asano, Koji Takada, Tamami Morisaki, Katsuyuki Takahashi, Hisakazu Fujita, Masatsune Shibutani, Ryosuke Amano, Tsutomu Takashima, Shuhei Tomita, Kosei Hirakawa, Masaichi Ohira

**Affiliations:** 1 Department of Breast and Endocrine Surgery, Osaka City University Graduate School of Medicine, 1-4-3 Asahi-machi, Abeno-ku, Osaka, 545-8585 Japan; 2 Department of Pharmacology, Osaka City University Graduate School of Medicine, 1-4-3 Asahi-machi, Abeno-ku, Osaka, 545-8585 Japan; 3 Department of Scientific and Linguistic Fundamentals of Nursing, Osaka City University Graduate School of Nursing, 1-5-17 Asahi-machi, Abeno-ku, Osaka, 545-0051 Japan; 4 Department of Gastrointestinal Surgery, Osaka City University Graduate School of Medicine, 1-4-3 Asahi-machi, Abeno-ku, Osaka, 545-8585 Japan; 5 Department of Hepato-Biliary-Pancreatic Surgery, Osaka City University Graduate School of Medicine, 1-4-3 Asahi-machi, Abeno-ku, Osaka, 545-8585 Japan

**Keywords:** Iron chelator, Eribulin mesylate, Breast cancer, Xenograft, Epithelial-mesenchymal transition, Immune checkpoints

## Abstract

**Background:**

Iron is required for the proliferation of cancer cells, and its depletion suppresses tumor growth. Eribulin mesylate (eribulin), a non-taxane microtubule inhibitor, disrupts the tumor microenvironment via vascular remodeling and obstruction of the epithelial-mesenchymal transition (EMT). Herein, we investigated the effects of the iron chelator on tumor-related properties of breast cancer cells and the effects of iron chelator plus eribulin on tumor growth in vivo.

**Methods:**

Two triple-negative breast cancer (TNBC) cell lines, MDA-MB-231 and BT-549, and one hormone-receptor positive breast cancer cell line, MCF-7, were used in our study. Cell proliferation, cell migration, cell cycle position, and gene expression were analyzed via MTT assays, wound-healing assays, flow cytometry, and quantitative real-time-polymerase chain reaction, respectively. For the in vivo experiments, mice with breast cancer xenografts were treated with the inhibitors, alone or together, and tumor volume was determined.

**Results:**

Iron chelator inhibited breast cancer cell proliferation and decreased the proportion of S-phase cells. Conversely, it induced hypoxia, angiogenesis, EMT, and immune checkpoints, as determined by quantifying the expression of marker mRNAs in MDA-MB-231 and MCF-7 cells. Eribulin suppressed the expression of the hypoxia and EMT related marker mRNAs in the presence of iron chelator. Iron chelator plus eribulin inhibited tumor growth in vivo to a greater extent than did either inhibitor alone.

**Conclusions:**

Although iron chelator induces oncogenic events (hypoxia, angiogenesis, EMT, and immune checkpoints), it may be an effective treatment for breast cancer when administered in combination with eribulin.

**Supplementary Information:**

The online version contains supplementary material available at 10.1186/s12885-020-07673-9.

## Background

Iron is an essential requirement for both normal and cancer cell viability and proliferation. The protein transferrin shuttles iron from the serum to the cell interior via transferrin receptor 1 (TfR1) [1]. Cancer cells have significantly higher levels of TfR1 than do normal cells and hence take up iron more rapidly [2]. Iron has been shown to increase the expression of cyclin D1, D2, and D3, which facilitates G1/S cell cycle progression [3], and is distributed in the hemoglobin of red blood cells, where it promotes oxygen transport. Conversely, iron depletion inhibits cyclin D1 expression [3, 4], suppresses tumor growth [5], and reduces serum hemoglobin levels and oxygen supply to tissues [6, 7]. These observations implicate iron in tumor progression; however, the effect of iron on the epithelial-mesenchymal transition (EMT) or immune checkpoints has not been examined sufficiently.

Eribulin mesylate (eribulin), a non-taxane, synthetic inhibitor of microtubule dynamics, induces G2/M cell cycle arrest and subsequent apoptosis [8–10]. Interestingly, it also has some unique anticancer effects in breast cancer cells, such as improvement of tumor perfusion, hypoxia [11], and the EMT [12]. We previously investigated these effects using clinical tumor samples [13] and suggested that eribulin enhanced the antitumor immune response by inactivating immune checkpoints [14]. The present study investigated the mechanism of iron control therapy in breast cancer in terms of the tumor microenvironment. We also determined whether the combination of iron depletion and eribulin was a potentially effective treatment for breast cancer.

## Methods

### Cell lines and culture conditions

Two triple-negative breast cancer (TNBC) cell lines, MDA-MB-231 and BT-549, and one hormone-receptor positive breast cancer (HRBC) cell lines, MCF-7, were from the American Type Culture Collection (Rockville, MD, USA). Cells were cultured in Dulbecco’s Modified Eagle’s Medium (DMEM; Wako, Osaka, Japan) supplemented with 10% fetal bovine serum (FBS; Equitech-Bio, Kerrville, TX, USA), 100 U/mL penicillin (Gibco, Grand Island, NE, USA), and 100 μg/mL streptomycin at 37 °C in humidified air with 5% CO_2_. The culture medium was replaced every 3 days.

### Compounds

Deferoxamine (DFO), an iron-chelating agent, was purchased from Novartis (Basel, Switzerland). Deferasirox, an another iron-chelator, was purchased from Novartis Pharma (Basel, Switzerland). Eribulin was provided by Eisai Co. (Tokyo, Japan).

### Cytotoxicity assay

The sensitivity of the three human breast cancer cell lines to two iron chelators was evaluated using a cell proliferation (MTT) assay kit according to the manufacturer’s instructions (Sigma-Aldrich, St. Louis, MO, USA). Briefly, cells (1 × 10^3^ cells/well in 96-well plates) were cultured for 24 h in 90 μL of DMEM with 10% FBS. A 10-μL aliquot of medium containing the indicated concentration of DFO or deferasirox was then added to each well. 72 h after incubation, 10 μL of the MTT reagent was added to each well; 2 h later, the medium was discarded and replaced by 100 μL of dimethyl sulfoxide. After shaking the plates for 10 min, the plate was measured as absorbance at 510 nm with a microplate reader (Perkin-Elmer, Waltham, MA, USA). Three independent experiments were performed [15].

### Wound-healing assay

MDA-MB-231 cells were cultured in 96-well plates. After the cells reached 80–90% confluence, a wound was created in the cell monolayer using the WoundMaker tool (Essen Bioscience, Ann Arbor, MI, USA). The cells were cultured in DMEM with 1% FBS and 10 or 100 μM DFO for 30 h. Scratched fields were photographed every 2 h using an Incucyte Live-Cell Imaging System (Essen Bioscience). The degree of cell migration was calculated as 100 × the wound closure area at each time point/the wound area at time 0 [16].

### Analysis of cell cycle progression

Cells (1 × 10^6^ cells/well) were plated into six-well tissue culture plates. After 24 h, the cells were harvested and washed two times with phosphate-buffered saline and stained with the CycleTEST™ PLUS DNA Reagent kit according to the manufacturer’s instructions (Becton Dickinson Biosciences, CA-San Jose, USA). Staining intensity was quantified using BD LSR II flow cytometer with FACSDiva™ software (Becton Dickinson Biosciences) [17, 18].

### Quantitative real-time-polymerase chain reaction (qRT-PCR)

Total RNA was extracted from cells using the RNeasy Mini kit (Qiagen, Valencia, CA, USA). cDNA was synthesized using ReverTra Ace qPCR RT Master Mix (TOYOBO, Osaka, Japan). The RT step was performed at 37 °C for 15 min, 50 °C for 5 min, and then 98 °C for 5 min. cDNA was amplified via qRT-PCR with Taq DNA polymerase (Nippon Gene, Tokyo, Japan) and the StepOnePlus RT-PCR system (Applied Biosystems, Foster City, CA, USA). The following TaqMan gene expression assays for used*:* assay ID, Hs00154208 (*CA9*); assay ID, Hs00911700 (*KDR*); assay ID, Hs00983056 (*CDH2*); assay ID, Hs00232783 (*ZEB1*); assay ID, Hs01125301 (*CD274*); and assay ID, Hs02758991 (*GAPHD*) (Applied Biosystems). RT-PCR was performed at 95 °C for 15 s, followed by 40 cycles at 60 °C for 60 s [15].

### In vivo tumor model

In vivo experiments were conducted on 4-week-old female athymic BALB/c nu/nu mice obtained from CLEA Japan (Tokyo, Japan). The animal experimental protocol was approved by Ethical Committee of the Osaka City University, Osaka, Japan. All studies on mice were conducted in accordance with the National Institute of Health (NIH) ‘Guide for the Care and Use of Laboratory Animals’. The mice were housed in a standard animal laboratory with ad libitum access to food and water. MDA-MB-231 cells (10^7^ cells) were suspended in 100 μL of DMEM, and injected into the backs of the mice. After a week, the mice were randomized into two groups: (1) control (saline alone) and (2) DFO (20 mg/kg/day, 5 days/week). For evaluation of the combination therapy, the mice were randomized into four groups: (1) control (saline alone), (2) DFO alone, (3) eribulin alone (0.8 mg/kg/day, 5 days/week), and (4) DFO plus eribulin.

Both DFO and eribulin were dissolved in DMEM without FBS. DFO was directly injected into the tumor, and eribulin was intravenously administered. The resultant tumor volumes (length × width) were measured weekly. Animals were euthanized via isoflurane (Mylan, PA, USA) (1 ml per one mouse) and cervical dislocation, and autopsied at 6 weeks after cell inoculation [17]. In sacrifice, animals were unconscious.

### Statistical analysis

Statistical analyses were performed with JMP13 software (SAS Institute, Cary, NC, USA). Student’s t-test was used to compare data between groups. A *p* value < 0.05 was considered statistically significant.

## Results

### Effects of iron chelator on breast cancer cell proliferation and migration

**Fig. 1:**
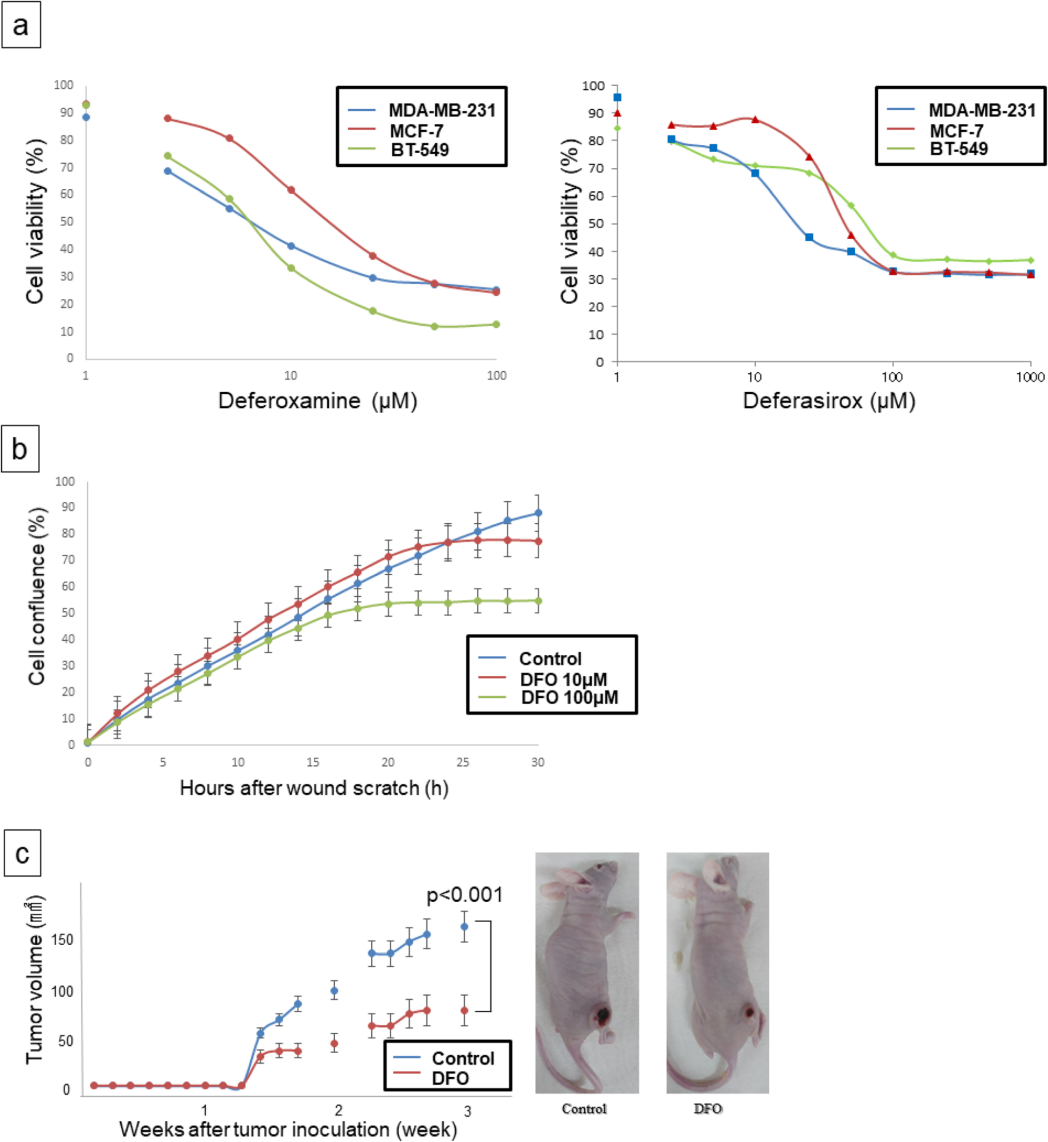
Cytotoxicity of deferoxamine and deferasirox (**a**). The sensitivity of MDA-MB-231, BT-549 and MCF-7 cell lines to deferoxamine was evaluated via an MTT assay. Deferoxamine and deferasirox suppressed cell proliferation of breast cancer cells in a dose-dependent manner. Effect of deferoxamine on the migration of breast cancer cells (**b**). The degree of MDA-MB-231 cells migrating across the wound did not change significantly by deferoxamine administration compared with the control. Tumor volumes of MDA-MB-231 cells xenograft in the group of control and deferoxamine administration in mice (tumor volume: control vs. iron-deficient = 159.9 ± 14.7 mm^2^ vs. 58.5 ± 12.1 mm^2^; *p* < 0.001) (**c**). The results of this analysis showed that the size of MDA-MB-231xenografts in the iron-deficient group was smaller than those in control group

**Fig. 2:**
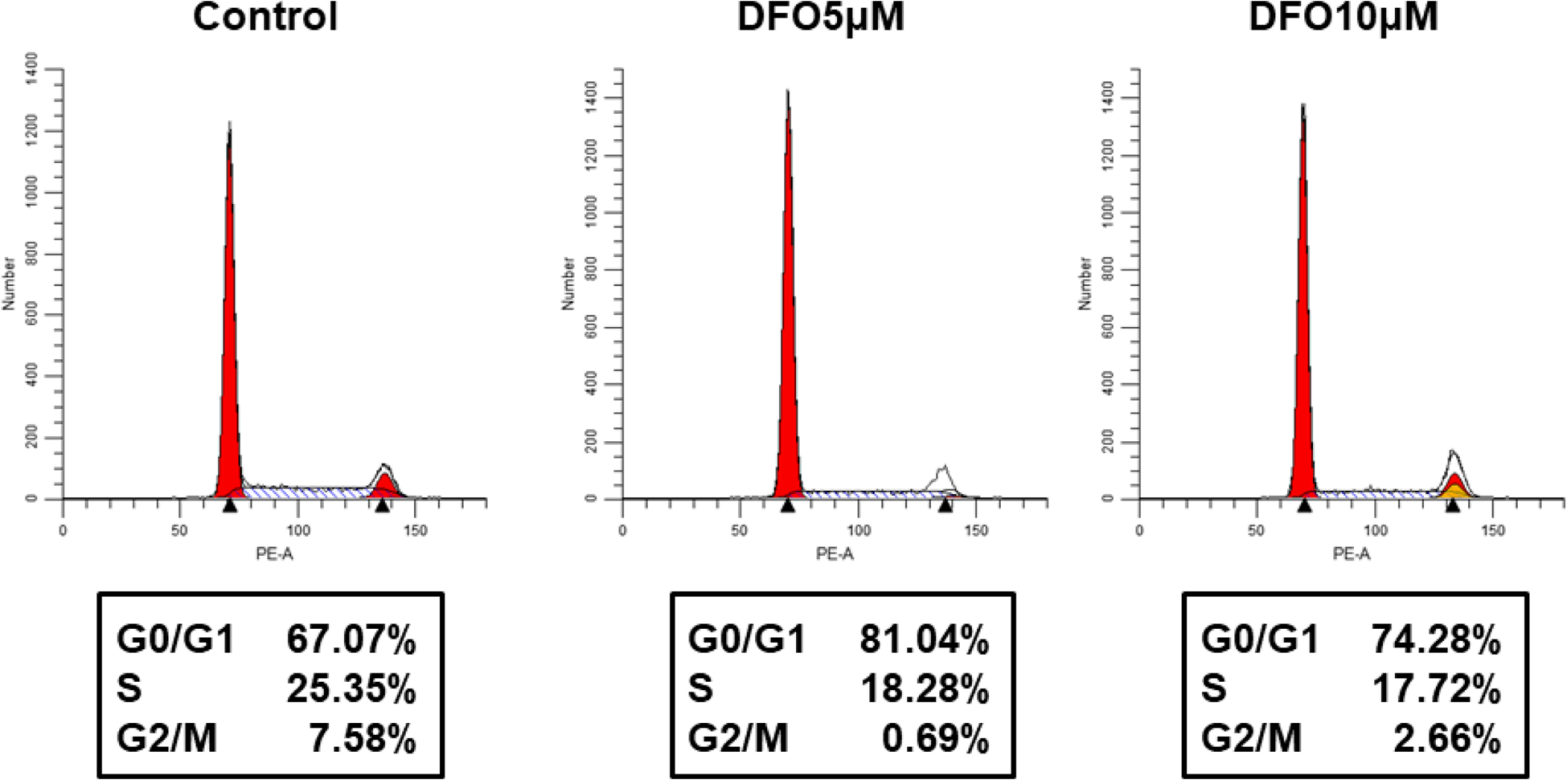
Cell cycle analysis of MDA-MB-231 and MDA-MB-231 + deferoxamine. The results of this analysis showed that the proportion of MDA-MB-231 cells that were in G0/G1 was decreased, after deferoxamine treatment

DFO and deferasirox suppressed the proliferation of all breast cancer cell lines in a dose-dependent manner in MTT assays (Fig. 1a). Wound healing assays showed that treatment with DFO inhibited the proliferation of MDA-MB-231 breast cancer cells but not the migration (Fig. 1b). DFO also suppressed tumor growth in nude mice with subcutaneous MDA-MB-231 xenografts (tumor volume: control vs. iron-deficient = 159.9 ± 14.7 mm^2^ vs. 58.5 ± 12.1 mm^2^; *p* < 0.001) **(**Fig. 1c**)**. No significant side effects were observed in DFO-treated mice. As shown via flow cytometry, DFO reduced the proportion of MDA-MB-231 cells in S phase **(**Fig. 2**)**. This finding suggests that iron depletion inhibits the G1/S transition.

### Effects of DFO on mRNA expression in breast cancer cells

In these experiments, cells were treated with or without DFO or deferasirox for 3 days. Total RNA was extracted, and the expression levels of the indicated mRNAs were quantified via qRT-PCR.

**Fig. 3:**
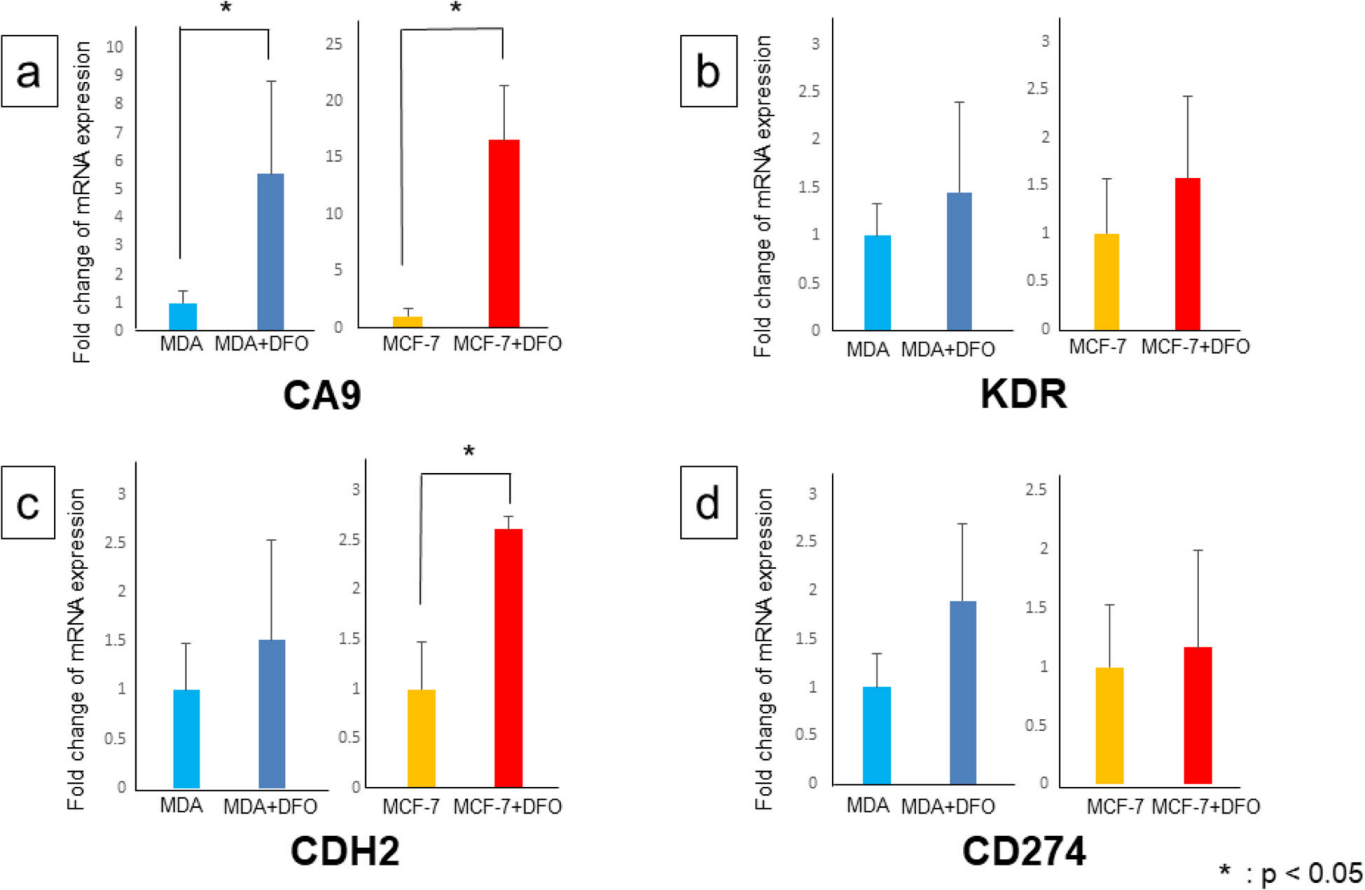
Expression levels of CA9 related gene in deferoxamine-treated MDA-MB-231 and MCF-7 cells as measured by RT-PCR (**a**). DFO upregulated *CA9* expression in both MDA-MB-231 and MCF-7 cells, indicating that iron deficiency induces hypoxia. Expression levels of VEGFR2 related gene in deferoxamine-treated MDA-MB-231 and MCF-7 cells as measured by RT-PCR (**b**). Deferoxamine treatment increased *KDR* expression levels in MDA-MB-231 and MCF-7 cells. Expression levels of N-cadherin related gene in deferoxamine-treated MDA-MB-231 and MCF-7 cells as measured by RT-PCR (**c**). Deferoxamine treatment increased *CDH2* expression levels in MDA-MB-231 and MCF-7 cells. Expression levels of PD-L1 related gene in deferoxamine-treated MDA-MB-231 and MCF-7 cells as measured by RT-PCR (**d**). Deferoxamine treatment increased *CD274* expression levels in MDA-MB-231 and MCF-7 cells

The effects of iron depletion on hypoxia and angiogenesis were examined by measuring the mRNA levels of *CA9* and *KDR*, respectively. *CA9* encodes carbonic anhydrase 9, which is overexpressed in hypoxic conditions, and *KDR* encodes vascular endothelial growth factor receptor 2, which promotes angiogenesis. Notably, DFO upregulated *CA9* expression in MDA-MB-231 and MCF-7 cells, indicating that iron deficiency induces hypoxia **(**Fig. 3a**)**. It also slightly increased the mRNA levels of *KDR* in MDA-MB-231 and MCF-7 **(**Fig. 3b**)**.

The effects of iron deficiency by DFO on the EMT and immune checkpoints were assessed by measuring the mRNA levels of *CDH2* and *CD274*, respectively. *CDH2* encodes N-cadherin, which is expressed by mesenchymal cells, and *CD274* encodes programmed death-ligand 1 (PD-L1), which activates an immune checkpoint. *CDH2* expression levels were higher in DFO-treated MDA-MB-231 and MCF-7 cells than in untreated cells **(**Fig. 3c**)**, as were *CD274* expression levels **(**Fig. 3d**)**.

**Fig. 4:**
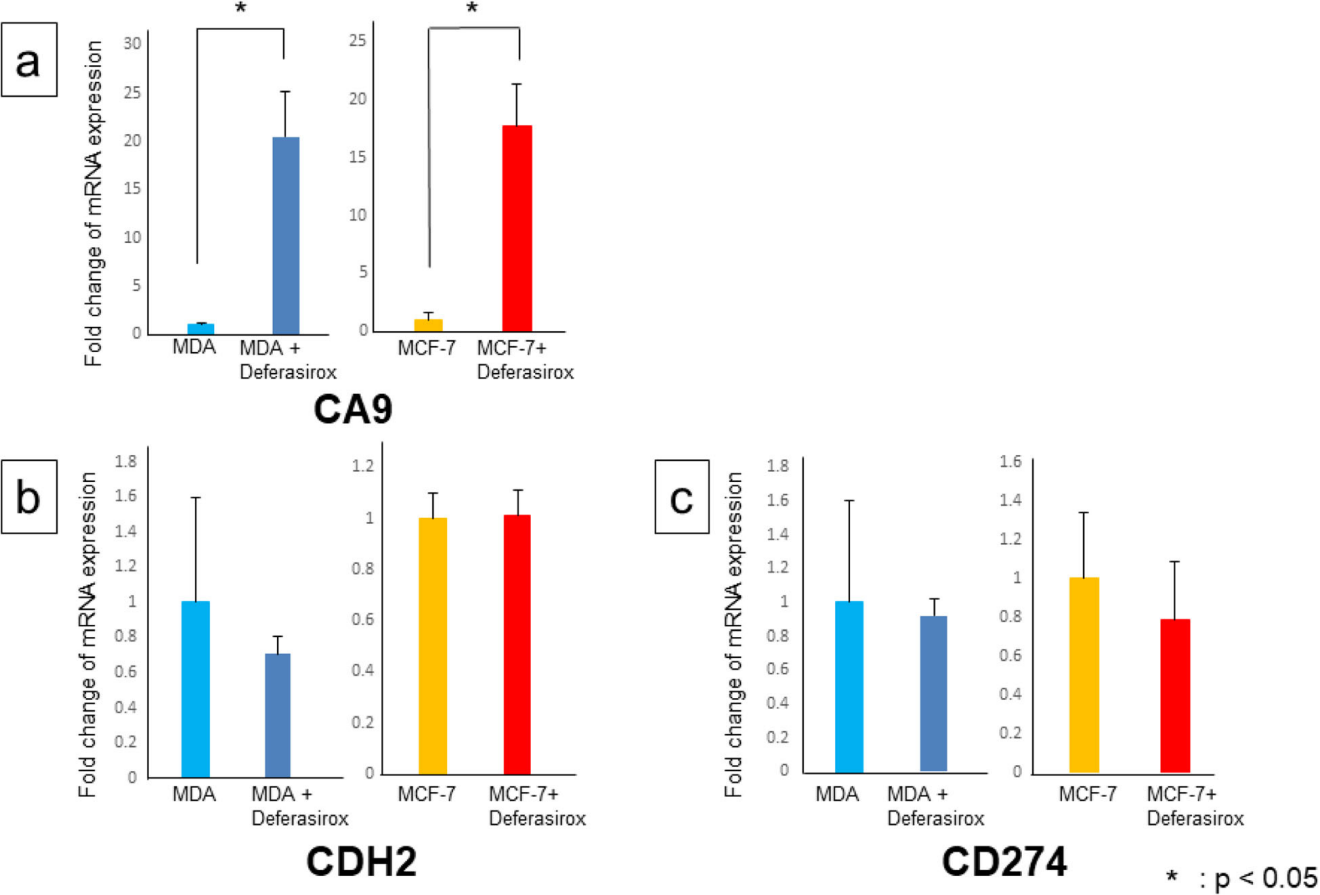
Expression levels of *CA9* related gene in deferasirox-treated MDA-MB-231 and MCF-7 cells as measured by RT-PCR (**a**). Deferasirox upregulated *CA9* expression in both MDA-MB-231 and MCF-7 cells, indicating that iron deficiency induces hypoxia. Expression levels of N-cadherin related gene in deferasirox-treated MDA-MB-231 and MCF-7 cells as measured by RT-PCR (**b**). Deferasirox treatment did not significantly change *CDH2* expression levels in MDA-MB-231 and MCF-7 cells. Expression levels of PD-L1 related gene in deferasirox-treated MDA-MB-231 and MCF-7 cells as measured by RT-PCR (**c**). Deferasirox treatment did not change *CD274* expression levels in MDA-MB-231 and MCF-7 cells

On the other hand, deferasirox also significantly upregulated *CA9* expression in MDA-MB-231 and MCF-7 cells (Fig. 4a). However, administration of deferasirox did not significantly change the expression of *CDH2* and *CD274* in both MDA-MB-231 and MCF-7 cells (Fig. 4b, c).

### Effects of eribulin plus DFO on tumor growth in mice

**Fig. 5:**
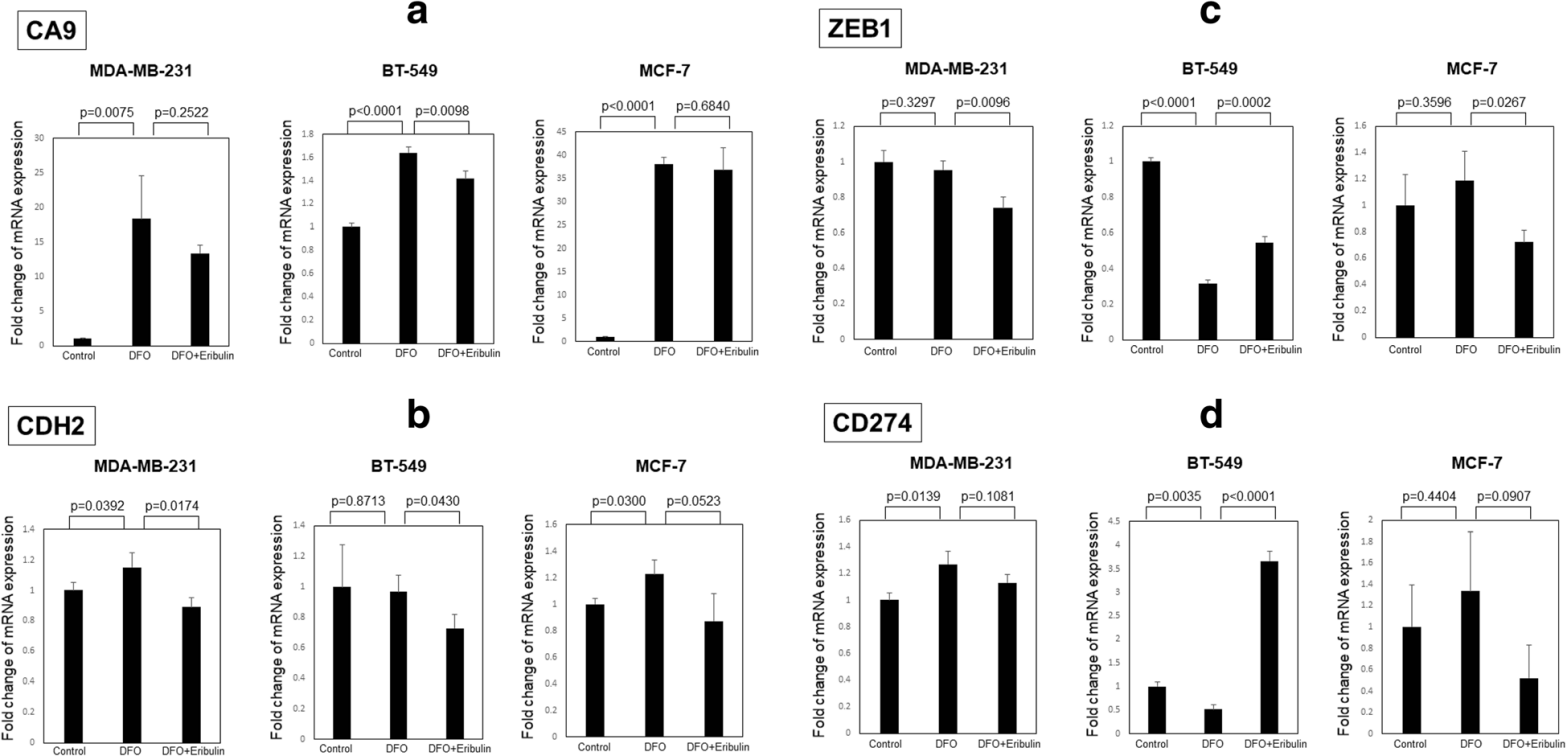
Expression levels of CA9 related gene in MDA-MB-231, BT-549 and MCF-7 cells treated with combination therapy of eribulin and deferoxamine as measured by RT-PCR (**a**). Eribulin significantly downregulated *CA9* expression levels in iron-deficient BT-549 cells by treated with deferoxamine (*p* = 0.0098). Expression levels of N-cadherin related gene, *CDH2* (**b**) and *ZEB1* (**c**), in MDA-MB-231, BT-549 and MCF-7 cells treated with combination therapy of eribulin and deferoxamine as measured by RT-PCR. Eribulin significantly downregulated *CDH2* expression levels in iron-deficient MDA-MB-231 and BT-549 cells by treated with deferoxamine (*p* = 0.0174, *p* = 0.0430, respectively), and significantly downregulated *ZEB1* expression levels in iron-deficient MDA-MB-231 and MCF-7 cells by treated with deferoxamine (*p* = 0.0096, *p* = 0.0267, respectively). Expression levels of PD-L1 related gene in MDA-MB-231, BT-549 and MCF-7 cells treated with combination therapy of eribulin and deferoxamine as measured by RT-PCR (**d**). Eribulin slightly downregulated *CD274* expression levels in iron-deficient MDA-MB-231 and MCF-7 cells by treated with deferoxamine

**Fig. 6:**
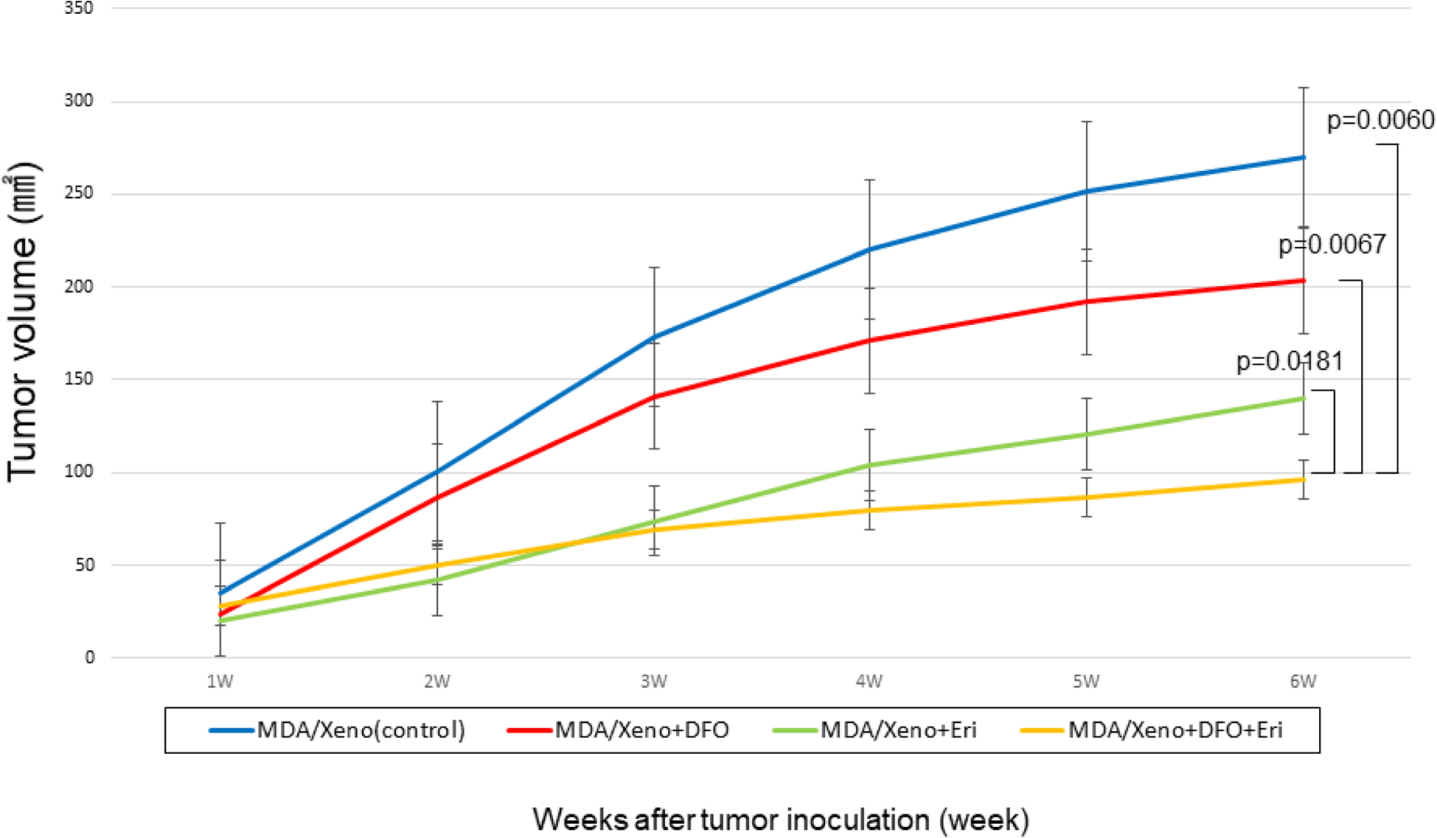
Tumor volumes of MDA-MB-231 cells xenograft in the group of control, deferoxamine alone, eriublin alone and combination of eribulin and deferoxamine in mice (tumor volume: control [270.0 ± 35.7 mm^2^], iron-deficient [203.5 ± 33.2 mm^2^], eribulin [141.9 ± 15.1 mm^2^], iron-deficient + eribulin [96.3 ± 13.8 mm^2^]) (*p* = 0.0181, *p* = 0.0067, *p* = 0.0060, respectively). The results of this analysis showed that tumor growth of MDA-MB-231cells xenografts in the combination treatment group was inhibited compared to eribulin alone or deferoxamine alone group

Our data suggest that DFO has both anti-oncogenic (inhibition of proliferation) and oncogenic (induction of hypoxia, EMT, and immune checkpoints) effects. Because we have shown that eribulin blocks these oncogenic events [13, 14], we asked whether DFO might more effectively suppress tumor growth in the presence of eribulin. Eribulin reduced *CA9* expression in the presence of DFO, especially in BT-549 cells **(**Fig. 5a**)**. In MDA-MB-231 cell lines, eribulin also significantly reduced expression of EMT related mRNA, *CDH2* and *ZEB1*, in the presence of DFO **(**Fig. 5b,c**)**. Regarding immune check points, eribulin did not significantly suppress CD274 expression in the presence of DFO **(**Fig. 5d**)**. Importantly, DFO plus eribulin inhibited the growth of MDA-MB-231 xenografts in nude mice to a greater extent than did either agent alone (tumor volume: control [270.0 ± 35.7 mm^2^], iron-deficient [203.5 ± 33.2 mm^2^], eribulin [141.9 ± 15.1 mm^2^], iron-deficient + eribulin [96.3 ± 13.8 mm^2^]) (*p* = 0.0181, *p* = 0.0067, *p* = 0.0060, respectively) **(**Fig. 6**)**. HE staining showed that vessels were increased in MDA-MB-231 xenograft tissue treated with DFO. On the other hand, in the xenograft treated with DFO plus eribulin, vessels were not increased (Supplementary Fig. 1).

## Discussion

**Fig. 7:**
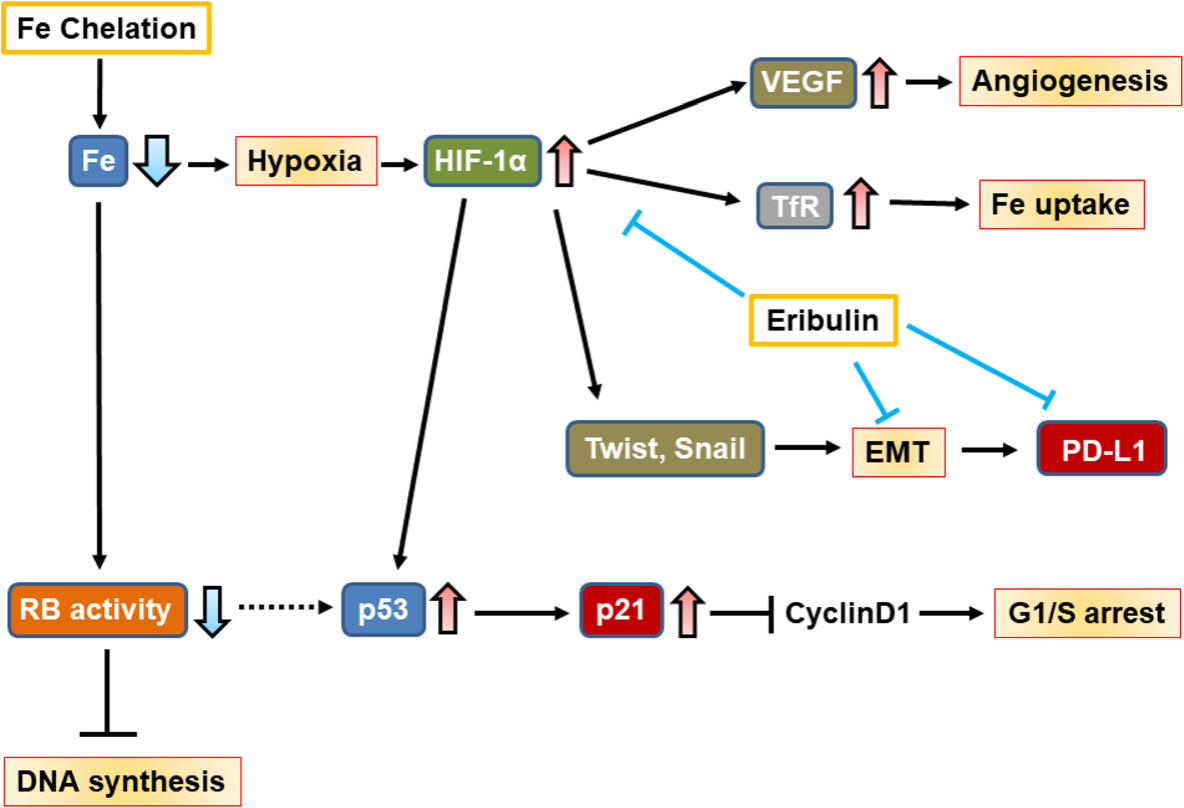
Schematic illustration of the effects of iron chelation and eribulin. Iron depletion can inhibit ribonucleotide reductase (RR) activity that results in suppressed DNA synthesis. Importantly, Iron depletion induces hypoxia and consequently promotes the expression of HIF-1α. HIF-1α can initiate the transcription of genes involved in angiogenesis and iron uptake, namely, VEGF and TfR, respectively. Iron chelation can also result in elevated p53 protein levels via the HIF-1α. Increased p53 stability then results in G1/S arrest and apoptosis via downstream effectors including p21. Furthermore, HIF-1α mediates EMT by upregulating Snail and TWIST1 expression. Increased p53 also induce various EMT transcription factors including ZEB-1 and miR200, and these factors upregulate PD-L1 expression. Eribulin treatment improves tumor immune microenvironment such as hypoxia, EMT and immune check point induced by iron depletion

In the present study, the iron chelator DFO suppressed breast cancer cell proliferation and tumor growth. In addition, iron-depleted condition may induce angiogenesis, hypoxia, EMT, and immune checkpoints. Regarding mechanism, we suggest that DFO promotes angiogenesis via hypoxia. To the best of our knowledge, our study is the first to examine the effects of iron depletion on the tumor immune microenvironment. Our model whereby DFO elicits its various effects is presented in Fig. 7.

As shown in a previous study, DFO induces hypoxia and the consequent expression of hypoxia-inducible factor-1 alpha (HIF-1α) in breast cancer cells [19]. HIF-1α initiates the transcription of genes encoding angiogenesis-promoting proteins such as vascular endothelial growth factor [20]. It also increases TfR expression, stimulates iron uptake [2], and interacts with and stabilizes the tumor suppressor p53 [21, 22]. Once accumulated, p53 may induce G1/S arrest and apoptosis via its downstream effectors [23].

Although several factors promote the EMT via complex pathways, HIF-1α is considered to be one of the most important [24]; it upregulates two EMT inducers, Snail and TWIST1 [25]. Hypoxia-induced HIFα has also been linked to immune checkpoint activation: in a previous study, it increased the expression of PD-L1 in myeloid-derived suppressor cells [26]. Furthermore, as shown by Noman et al., PD-L1 expression is upregulated in EMT-activated breast cancer cells, a process driven by various EMT transcription factors, including ZEB-1 and miR200 [27]. Based on these findings, we suggest that iron depletion induces the EMT and immune checkpoints via HIF-1α.

Because it elicits oncogenic events (hypoxia, EMT, and immune checkpoints), as well as anti-oncogenic events (G1/S arrest and apoptosis), iron depletion monotherapy is considered insufficient for the treatment of breast cancer. Hence, recent studies assessed the effectiveness of iron depletion plus chemotherapy. Hoke et al. found that the combination of DFO as an antioxidant and doxorubicin improved the outcomes of breast cancer patients, perhaps by reducing the toxicity of doxorubicin to cardiomyocytes and tumor growth [28]. Moreover, Ohara et al. reported that the combination of iron depletion and bevacizumab, an antiangiogenic drug, had dramatic synergistic antitumor effects [17].

Eribulin is currently an approved treatment for patients with locally advanced or metastatic breast cancer. In a phase III trial (study 305/EMBRACE), eribulin significantly improved the overall survival of metastatic breast cancer patients who had previously undergone chemotherapy with anti-cancer agents such as anthracycline and taxane [29]. Since recent studies indicate that eribulin inhibits tumor hypoxia, EMT, and immune checkpoints [11–14], we hypothesized that iron depletion plus eribulin might be a useful therapy for breast cancer. No previous studies have investigated this combination therapy in breast cancer.

We found that the combination of DFO and eribulin had a significant antitumor effect in vivo compared with either inhibitor alone. Additionally, our qRT-PCR results suggest that eribulin more or less suppressed the induction of hypoxia, EMT, and immune checkpoints by DFO especially in MDA-MB-231 and MCF-7 cells. We previously used tumor-infiltrating lymphocytes to monitor the tumor immune microenvironment and propose that doing so is a valid means for evaluating the therapeutic effects of eribulin in TNBC cases [30]. This study showed that the effects of eribulin was associated with immune microenvironment. Our present study suggests that eribulin might act synergistically with DFO to suppress tumor growth in activated tumor immune microenvironments, such as those produced by DFO. Hence, eribulin plus iron depletion potentially represents a novel and practical treatment for breast cancer. However, eribulin incompletely suppressed the expression of the marker mRNAs for hypoxia, EMT, and immune checkpoints in DFO-treated cells (i.e., expression levels were higher in cells receiving eribulin plus DFO than in those receiving eribulin alone). Consequently, the effect of the combination on tumor suppression may be additive, not synergistic. In addition, even in the same TNBC cell lines, the change of *ZEB1* and *CD274* expression in BT-549 cells were different from MDA-MB-231. These results suggest that the mechanism of tumor immune microenvironment underlying iron deficiency may differ in various breast cancer cell lines. Moreover, according to deferasirox, another iron chelator, some previous studies revealed that deferasirox was noninferior or more effective than DFO regarding the iron removal [31, 32]. In present study, though deferasirox showed inhibition of breast cancer proliferation and induction of hypoxia like DFO, it did not show the similar result with respect to EMT or immune check points. One of the possible reasons for these inconsistent results of in vitro experiments may be that DFO is an injection while deferasirox is an oral drug. Hence, further studies are necessary to decipher the complex mechanisms underlying the effects of eribulin and DFO on tumor suppression and to design strategies for DFO and eribulin cotreatment of breast cancer patients in the clinical setting.

## Conclusions

Iron depletion inhibits the proliferation and in vivo growth of breast cancer cells. Conversely, it induces hypoxia, EMT, and immune checkpoints in some breast cancer cell lines, thus creating an environment conducive for tumor growth. The combination of DFO and eribulin may be an effective treatment for breast cancer.

## Supplementary Information

**Additional file 1: Supplementary Fig. 1** HE staining showed that vessels (arrow) were increased in the iron-deficient condition. However, vessels were not increased in the tumor treated with combination therapy, DFO plus eribulin.

## Data Availability

The datasets used and/or analyzed during the current study are available from the corresponding author on reasonable request.

## Abbreviations

DFO: Deferoxamine
DMEM: Dulbecco’s modified Eagle’s medium
EMT: Epithelial-mesenchymal transition
FBS: Fetal bovine serum
NIH: National Institute of Health
PD-L1: Programmed death-ligand 1
qRT-PCR: Quantitative real-time-polymerase chain reaction
TfR1: Transferrin receptor 1
TNBC: Triple-negative breast cancer
