# Supplementary figures and images for: Inhibitory effects of iron depletion plus eribulin on the breast cancer microenvironment
Source: BMC Cancer. 2020 Dec 10;20:1215. doi: 10.1186/s12885-020-07673-9 (PMC7727180; doi:10.1186/s12885-020-07673-9)

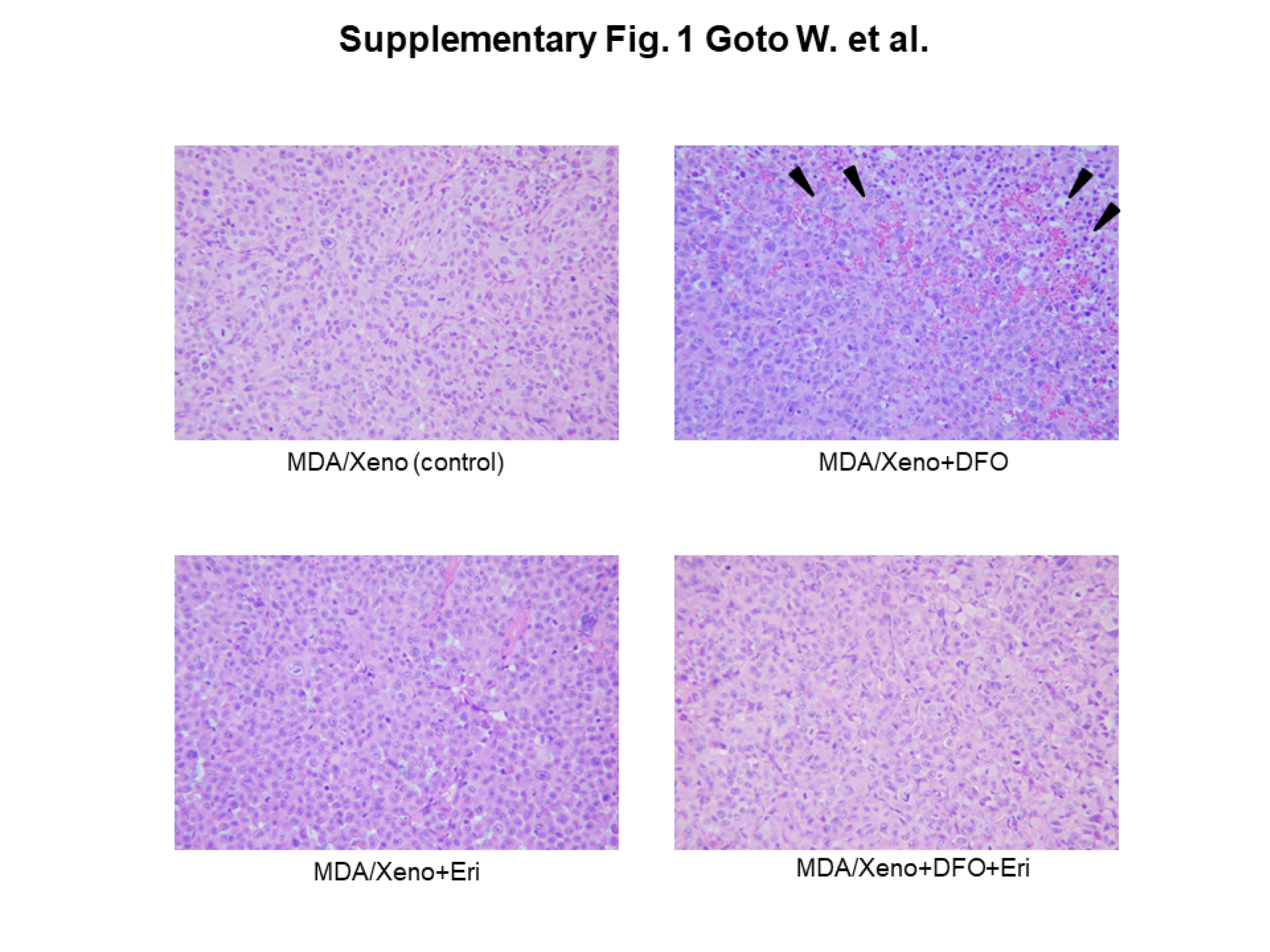

Supplement: Supplementary file 1 — Additional file 1: Supplementary Fig. 1 HE staining showed that vessels (arrow) were increased in the iron-deficient condition. However, vessels were not increased in the tumor treated with combination therapy, DFO plus eribulin. [file 12885_2020_7673_MOESM1_ESM.tif]
